# 5-Bromo-*N*-(3,4-dimeth­oxy­benz­yl)pyridin-2-amine

**DOI:** 10.1107/S1600536812015796

**Published:** 2012-04-18

**Authors:** Jie Li, Lin-Yan Lu, Wang-Xing Shen, Jian-You Shi

**Affiliations:** a School of Medicine and Life Sciences, Zhejiang University City College, Hangzhou 310015, Zhejiang, People’s Republic of China; b Sichuan Academy of Medical Sciences and Sichuan Provincial People’s Hospital, Chengdu, Sichuan 610072, People’s Republic of China

## Abstract

The title compound, C_14_H_15_BrN_2_O_2_, an inter­mediate in drug discovery, was synthesized by the reaction of 5-bromo­pyridin-2-amine and 3,4-dimeth­oxy­benzaldehyde. In the crystal, molecules are linked *via* pairs ofN—H⋯N hydrogen bonds, leading to the formation of inversion dimers. A short contact occurs between the aryl H atom (*ortho* position from N) and the centroid of the benzene ring.

## Related literature

For the anti-tumor activity of related compounds, see: Kovala-Demertzi *et al.* (2007 ▶). For the anti-ulcer activity of related compounds, see: Cho *et al.* (2001 ▶). For the anti-viral activity of related compounds, see: Mavel *et al.* (2002 ▶). For the anti-microbial activity of related compounds, see: Yeong *et al.* (2004 ▶).
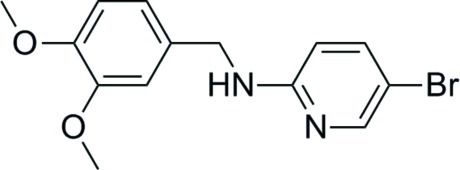


## Experimental

### 

#### Crystal data

C_14_H_15_BrN_2_O_2_
*M* *_r_* = 323.18
Monoclinic, 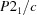

*a* = 6.3202 (2) Å
*b* = 13.7940 (4) Å
*c* = 15.8582 (6) Å
β = 100.961 (4)°
*V* = 1357.31 (8) Å^3^
*Z* = 4
Mo *K*α radiation
μ = 3.03 mm^−1^
*T* = 130 K
0.42 × 0.30 × 0.15 mm

#### Data collection

Agilent Xcalibur Eos diffractometer
Absorption correction: multi-scan (*CrysAlis PRO*; Agilent, 2011 ▶) *T*_min_ = 0.509, *T*_max_ = 1.000
8188 measured reflections
2384 independent reflections
2055 reflections with *I* > 2σ(*I*)
*R*_int_ = 0.030

#### Refinement

*R*[*F* ^2^ > 2σ(*F* ^2^)] = 0.027
*wR*(*F* ^2^) = 0.060
*S* = 1.03
2384 reflections
174 parameters
H-atom parameters constrained
Δρ_max_ = 0.47 e Å^−3^
Δρ_min_ = −0.49 e Å^−3^

### 

Data collection: *CrysAlis PRO* (Agilent, 2011 ▶); cell refinement: *CrysAlis PRO*; data reduction: *CrysAlis PRO*; program(s) used to solve structure: *SHELXS97* (Sheldrick, 2008 ▶); program(s) used to refine structure: *SHELXL97* (Sheldrick, 2008 ▶); molecular graphics: *OLEX2* (Dolomanov *et al.*, 2009 ▶); software used to prepare material for publication: *OLEX2*.

## Supplementary Material

Crystal structure: contains datablock(s) global, I. DOI: 10.1107/S1600536812015796/rk2346sup1.cif

Structure factors: contains datablock(s) I. DOI: 10.1107/S1600536812015796/rk2346Isup2.hkl

Supplementary material file. DOI: 10.1107/S1600536812015796/rk2346Isup3.cml

Additional supplementary materials:  crystallographic information; 3D view; checkCIF report

## Figures and Tables

**Table 1:** Hydrogen-bond geometry (Å, °) *Cg* is the centroid of the C7–C12 ring.

*D*—H⋯*A*	*D*—H	H⋯*A*	*D*⋯*A*	*D*—H⋯*A*
N2—H2⋯N1^i^	0.88	2.31	3.090 (3)	149
C2—H2*A*⋯*Cg*^i^	0.95	2.50	3.397 (3)	158

Symmetry code: (i) 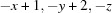.

**Fig. 1.**
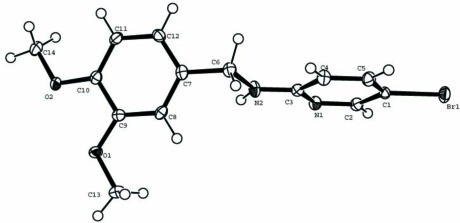
The molecular structure of title compound with the atom numbering scheme. Displacement ellipsoids are drawn at 50% probability level. H atoms are presented as a small spheres of arbitrary radius.

## supplementary crystallographic information

### Comment

The pyridine skeleton is a great importance to chemistry as well as biology which are well known for their versatile pharmacological activities such as anti-tumor (Kovala-Demertzi *et al.*, 2007), anti-ulcer (Cho *et al.*, 2001), anti-viral (Mavel *et al.*, 2002) and antimicrobial (Yeong *et al.*, 2004). The title compound is one of these compounds. The crystal packing is stabilized by a pair of strong intermolecular N2–H2···N1^i^ classical hydrogen bonds conecting two moleculars to form a centrosymmtric dimer. The H2A atom from pyridine moiety has short contact (2.50Å) with Cg^i^ of phenyl ring (C7-C12). Symmetry code: (i) 1-*x*, 2-*y*, -*z*.

### Experimental

A methanol solution of 5-bromopyridin-2-amine (1.73 g, 0.01 mol), 3,4-dimethoxybenzaldehyde (1.66 g, 0.01 mol) with sodium cyanoborohydride (0.69 g, 0.011 mol) was heated to reflux for 3 h. The mixture was poured into cold water and then filtered to get this compound. Single crystals were obtained from the powder in ethanol after 5 days.

### Refinement

H atoms were positioned geometrically (C–H = 0.95-0.99Å and N–H = 0.88Å) and refined using a riding model, with *U*_iso_(H) = 1.5*U*_eq_(C) for methyl groups and *U*_iso_(H) = 1.2*U*_eq_(C, N) for others.

### Crystal data

**Table:**

C_14_H_15_BrN_2_O_2_	*F*(000) = 656
*M**_r_* = 323.18	*D*_x_ = 1.582 Mg m^−^^3^
Monoclinic, *P*2_1_/*c*	Mo *K*α radiation, λ = 0.7107 Å
Hall symbol: -P 2ybc	Cell parameters from 3191 reflections
*a* = 6.3202 (2) Å	θ = 3.0–29.3°
*b* = 13.7940 (4) Å	µ = 3.03 mm^−^^1^
*c* = 15.8582 (6) Å	*T* = 130 K
β = 100.961 (4)°	Block, colourless
*V* = 1357.31 (8) Å^3^	0.42 × 0.30 × 0.15 mm
*Z* = 4	

### Data collection

**Table:**

Agilent Xcalibur Eos diffractometer	2384 independent reflections
Radiation source: Enhance (Mo) X-ray Source	2055 reflections with *I* > 2σ(*I*)
Graphite monochromator	*R*_int_ = 0.030
Detector resolution: 16.0874 pixels mm^-1^	θ_max_ = 25.0°, θ_min_ = 3.0°
ω–scans	*h* = −7→7
Absorption correction: multi-scan (*CrysAlis PRO*; Agilent, 2011)	*k* = −15→16
*T*_min_ = 0.509, *T*_max_ = 1.000	*l* = −18→15
8188 measured reflections	

### Refinement

**Table:**

Refinement on *F*^2^	Primary atom site location: structure-invariant direct methods
Least-squares matrix: full	Secondary atom site location: difference Fourier map
*R*[*F*^2^ > 2σ(*F*^2^)] = 0.027	Hydrogen site location: inferred from neighbouring sites
*wR*(*F*^2^) = 0.060	H-atom parameters constrained
*S* = 1.03	*w* = 1/[σ^2^(*F*_o_^2^) + (0.0214*P*)^2^ + 1.0069*P*] where *P* = (*F*_o_^2^ + 2*F*_c_^2^)/3
2384 reflections	(Δ/σ)_max_ = 0.001
174 parameters	Δρ_max_ = 0.47 e Å^−^^3^
0 restraints	Δρ_min_ = −0.49 e Å^−^^3^

### Special details

**Table:**

Experimental. Absorption correction: *CrysAlis Pro* (Agilent Technologies, 2011) Empirical absorption correction using spherical harmonics, implemented in SCALE3 ABSPACK scaling algorithm.
Geometry. All s.u.'s (except the s.u. in the dihedral angle between two l.s. planes) are estimated using the full covariance matrix. The cell s.u.'s are taken into account individually in the estimation of s.u.'s in distances, angles and torsion angles; correlations between s.u.'s in cell parameters are only used when they are defined by crystal symmetry. An approximate (isotropic) treatment of cell s.u.'s is used for estimating s.u.'s involving l.s. planes.
Refinement. Refinement of *F*^2^ against ALL reflections. The weighted *R*-factor *wR* and goodness of fit *S* are based on *F*^2^, conventional *R*-factors *R* are based on *F*, with *F* set to zero for negative *F*^2^. The threshold expression of *F*^2^ > σ(*F*^2^) is used only for calculating *R*-factors(gt) *etc*. and is not relevant to the choice of reflections for refinement. *R*-factors based on *F*^2^ are statistically about twice as large as those based on *F*, and *R*-factors based on ALL data will be even larger.

### Fractional atomic coordinates and isotropic or equivalent isotropic displacement parameters (Å^2^)

**Table:**

	*x*	*y*	*z*	*U*_iso_*/*U*_eq_	
Br1	0.29705 (4)	0.620125 (18)	0.137802 (17)	0.02180 (10)	
O1	0.8391 (3)	1.38453 (12)	0.14221 (11)	0.0181 (4)	
O2	1.1319 (3)	1.40582 (12)	0.05188 (11)	0.0185 (4)	
N1	0.4443 (3)	0.89466 (14)	0.06525 (13)	0.0164 (5)	
N2	0.7225 (3)	1.00200 (15)	0.10747 (13)	0.0178 (5)	
H2	0.6482	1.0443	0.0722	0.021*	
C1	0.4393 (4)	0.74052 (17)	0.13319 (15)	0.0148 (5)	
C2	0.3514 (4)	0.80926 (17)	0.07365 (16)	0.0165 (5)	
H2A	0.2181	0.7951	0.0368	0.020*	
C3	0.6333 (4)	0.91502 (18)	0.11831 (15)	0.0147 (5)	
C4	0.7300 (4)	0.84807 (18)	0.18126 (16)	0.0177 (5)	
H4	0.8617	0.8637	0.2186	0.021*	
C5	0.6323 (4)	0.76009 (18)	0.18814 (16)	0.0182 (6)	
H5	0.6958	0.7138	0.2297	0.022*	
C6	0.9380 (4)	1.02800 (18)	0.15245 (16)	0.0176 (6)	
H6A	1.0436	0.9803	0.1387	0.021*	
H6B	0.9432	1.0263	0.2152	0.021*	
C7	0.9971 (4)	1.12777 (17)	0.12650 (15)	0.0154 (5)	
C8	0.8867 (4)	1.20894 (18)	0.14866 (15)	0.0153 (5)	
H8	0.7767	1.2007	0.1814	0.018*	
C9	0.9355 (4)	1.30058 (17)	0.12366 (15)	0.0144 (5)	
C10	1.0981 (4)	1.31318 (18)	0.07442 (15)	0.0148 (5)	
C11	1.2084 (4)	1.23269 (18)	0.05377 (16)	0.0175 (6)	
H11	1.3198	1.2404	0.0217	0.021*	
C12	1.1576 (4)	1.14027 (18)	0.07967 (16)	0.0175 (6)	
H12	1.2342	1.0856	0.0649	0.021*	
C13	0.6735 (4)	1.37654 (19)	0.19176 (17)	0.0209 (6)	
H13A	0.5605	1.3329	0.1627	0.031*	
H13B	0.6116	1.4407	0.1979	0.031*	
H13C	0.7347	1.3506	0.2487	0.031*	
C14	1.2631 (4)	1.41893 (19)	−0.01160 (17)	0.0214 (6)	
H14A	1.2102	1.3772	−0.0611	0.032*	
H14B	1.4126	1.4018	0.0130	0.032*	
H14C	1.2564	1.4868	−0.0301	0.032*	

### Atomic displacement parameters (Å^2^)

**Table:**

	*U*^11^	*U*^22^	*U*^33^	*U*^12^	*U*^13^	*U*^23^
Br1	0.02543 (15)	0.01635 (15)	0.02437 (16)	−0.00657 (11)	0.00660 (11)	0.00055 (11)
O1	0.0203 (9)	0.0166 (9)	0.0204 (10)	0.0020 (7)	0.0109 (8)	0.0025 (8)
O2	0.0218 (9)	0.0140 (9)	0.0227 (10)	−0.0044 (7)	0.0116 (8)	−0.0004 (7)
N1	0.0156 (10)	0.0169 (11)	0.0153 (11)	−0.0021 (9)	−0.0002 (9)	0.0000 (9)
N2	0.0173 (10)	0.0137 (11)	0.0193 (12)	−0.0020 (9)	−0.0044 (9)	0.0042 (9)
C1	0.0196 (12)	0.0115 (13)	0.0146 (13)	−0.0018 (10)	0.0067 (11)	−0.0018 (10)
C2	0.0154 (12)	0.0180 (13)	0.0155 (14)	−0.0032 (10)	0.0017 (11)	−0.0036 (11)
C3	0.0146 (12)	0.0162 (13)	0.0131 (13)	−0.0010 (10)	0.0024 (10)	−0.0010 (10)
C4	0.0163 (12)	0.0185 (13)	0.0161 (14)	−0.0007 (10)	−0.0021 (11)	0.0030 (11)
C5	0.0194 (13)	0.0185 (14)	0.0167 (14)	0.0007 (11)	0.0037 (11)	0.0045 (11)
C6	0.0136 (12)	0.0178 (14)	0.0194 (14)	−0.0014 (10)	−0.0014 (11)	0.0006 (11)
C7	0.0145 (12)	0.0159 (13)	0.0135 (13)	−0.0030 (10)	−0.0034 (10)	−0.0010 (10)
C8	0.0136 (12)	0.0197 (14)	0.0124 (13)	−0.0030 (10)	0.0019 (10)	0.0018 (10)
C9	0.0121 (12)	0.0176 (13)	0.0129 (13)	0.0002 (10)	0.0008 (10)	−0.0018 (11)
C10	0.0138 (12)	0.0168 (13)	0.0129 (13)	−0.0033 (10)	0.0000 (10)	−0.0018 (10)
C11	0.0145 (12)	0.0213 (14)	0.0180 (14)	−0.0034 (11)	0.0061 (11)	−0.0018 (11)
C12	0.0157 (12)	0.0150 (14)	0.0212 (14)	0.0003 (10)	0.0018 (11)	−0.0039 (11)
C13	0.0179 (12)	0.0259 (15)	0.0206 (14)	0.0019 (11)	0.0076 (11)	−0.0013 (12)
C14	0.0215 (13)	0.0210 (14)	0.0236 (15)	−0.0048 (11)	0.0095 (12)	0.0014 (12)

### Geometric parameters (Å, º)

**Table:**

Br1—C1	1.897 (2)	C6—H6B	0.9900
O1—C9	1.366 (3)	C6—C7	1.504 (3)
O1—C13	1.427 (3)	C7—C8	1.399 (3)
O2—C10	1.355 (3)	C7—C12	1.377 (3)
O2—C14	1.432 (3)	C8—H8	0.9500
N1—C2	1.334 (3)	C8—C9	1.377 (3)
N1—C3	1.353 (3)	C9—C10	1.414 (3)
N2—H2	0.8800	C10—C11	1.383 (3)
N2—C3	1.350 (3)	C11—H11	0.9500
N2—C6	1.457 (3)	C11—C12	1.395 (3)
C1—C2	1.378 (3)	C12—H12	0.9500
C1—C5	1.384 (3)	C13—H13A	0.9800
C2—H2A	0.9500	C13—H13B	0.9800
C3—C4	1.411 (3)	C13—H13C	0.9800
C4—H4	0.9500	C14—H14A	0.9800
C4—C5	1.375 (3)	C14—H14B	0.9800
C5—H5	0.9500	C14—H14C	0.9800
C6—H6A	0.9900		
			
C9—O1—C13	117.18 (18)	C12—C7—C8	119.3 (2)
C10—O2—C14	116.51 (18)	C7—C8—H8	119.6
C2—N1—C3	118.3 (2)	C9—C8—C7	120.8 (2)
C3—N2—H2	119.0	C9—C8—H8	119.6
C3—N2—C6	122.0 (2)	O1—C9—C8	125.6 (2)
C6—N2—H2	119.0	O1—C9—C10	114.5 (2)
C2—C1—Br1	119.77 (18)	C8—C9—C10	119.8 (2)
C2—C1—C5	119.3 (2)	O2—C10—C9	115.4 (2)
C5—C1—Br1	120.93 (18)	O2—C10—C11	125.6 (2)
N1—C2—C1	123.2 (2)	C11—C10—C9	118.9 (2)
N1—C2—H2A	118.4	C10—C11—H11	119.7
C1—C2—H2A	118.4	C10—C11—C12	120.6 (2)
N1—C3—C4	121.1 (2)	C12—C11—H11	119.7
N2—C3—N1	116.5 (2)	C7—C12—C11	120.5 (2)
N2—C3—C4	122.4 (2)	C7—C12—H12	119.8
C3—C4—H4	120.2	C11—C12—H12	119.8
C5—C4—C3	119.5 (2)	O1—C13—H13A	109.5
C5—C4—H4	120.2	O1—C13—H13B	109.5
C1—C5—H5	120.7	O1—C13—H13C	109.5
C4—C5—C1	118.5 (2)	H13A—C13—H13B	109.5
C4—C5—H5	120.7	H13A—C13—H13C	109.5
N2—C6—H6A	109.6	H13B—C13—H13C	109.5
N2—C6—H6B	109.6	O2—C14—H14A	109.5
N2—C6—C7	110.42 (19)	O2—C14—H14B	109.5
H6A—C6—H6B	108.1	O2—C14—H14C	109.5
C7—C6—H6A	109.6	H14A—C14—H14B	109.5
C7—C6—H6B	109.6	H14A—C14—H14C	109.5
C8—C7—C6	120.1 (2)	H14B—C14—H14C	109.5
C12—C7—C6	120.6 (2)		

### Hydrogen-bond geometry (Å, º)

Cg is the centroid of the C7–C12 ring.

**Table:**

*D*—H···*A*	*D*—H	H···*A*	*D*···*A*	*D*—H···*A*
N2—H2···N1^i^	0.88	2.31	3.090 (3)	149
C2—H2*A*···*Cg*^i^	0.95	2.50	3.397 (3)	158

Symmetry code: (i) −*x*+1, −*y*+2, −*z*.
